# Left atrial appendage occlusion in chronic kidney disease: opening the way to randomized trials

**DOI:** 10.1093/europace/euad342

**Published:** 2023-11-14

**Authors:** Odysseas Katsaros, Anastasios Apostolos, Konstantinos Toutouzas

**Affiliations:** First Department of Cardiology, National and Kapodistrian University of Athens, Hippokration General Hospital of Athens, 114 Vasilissis Sofias, Athens 115 27, Greece; First Department of Cardiology, National and Kapodistrian University of Athens, Hippokration General Hospital of Athens, 114 Vasilissis Sofias, Athens 115 27, Greece; First Department of Cardiology, National and Kapodistrian University of Athens, Hippokration General Hospital of Athens, 114 Vasilissis Sofias, Athens 115 27, Greece

We read with great interest the multicentre cohort study of Della Rocca *et al*.^1^ titled ‘Prognostic Value of Chronic Kidney Disease in Patients Undergoing Left Atrial Appendage Occlusion’ performed in order to evaluate the prognostic value of chronic kidney disease (CKD) in patients undergoing percutaneous left atrial appendage occlusion (LAAO). We would like to congratulate the authors on the outcomes of this multi-centre study with long-term results.

The study shows that nearly half of the patients (48.7%, *n* = 1035) having undergone LAAO have impaired renal function. Considering this, as well as the fact that these patients have a high ischaemic and haemorrhagic risk due to this impairment,^2,3^ it is highly significant to contemplate the renal function in those individuals. Although ischaemic and bleeding events were more frequent in CKD patients compared to the normal-renal-function-reference group, LAAO was still beneficial.

It would be very engaging and would furthermore add value to the results and discussion of this paper, to compare LAAO vs. medical treatment in patients with very low estimated glomerular filtration rate (eGFR). Patients who have overt CKD have more adverse effects and perhaps greater efficacy from LAAO—than those without CKD. Nevertheless, in these patients we have to compare the two different treatments they may receive. Moreover, patients with very low and extremely low eGFR [CKD stage 4 (8.0%, *n* = 170) and CKD stage 5 (3.2%, *n* = 69)] are unable to be treated with direct oral anticoagulants (DOAC) and dose of acenocoumarol should be modified, as well as their labile international normalized ratio (INR) can make its maintenance at the desired levels challenging.^4–6^ In that manner, we consider that LAAO may have further benefits, despite the numerical increase in peri-procedural complications compared to the reference arm, which is anticipated due to more comorbidities. Thus, such patients could have a much greater benefit from LAAO in terms of bleeding and ischaemic events, compared to medical treatment *per se*.

Furthermore, these findings show that improved renal function is associated with less peri-procedural complications, better efficacy, and safety of LAAO. Considering that CKD is a progressive, worsening disease, timely LAAO in earlier stages might be more beneficial. How useful would it also be to establish a risk score system for stroke prevention through LAAO, based on specific factors, by targeting either purely the CKD population or—in a more general score—the value of the CKD stage in the calculation.

We estimate that there is more to construe and based on the absence of potent concordant data from randomized control trials, we believe that the multi-centre randomized clinical trial LAA-KIDNEY (LAAO in Patients With Non-valvular Atrial Fibrillation and End-stage Chronic KIDNEY Disease; NCT05204212) is setting foot on randomized control trials comparing the efficacy of LAAO vs. best medical care in patients with CKD, that are just around the corner.
